# Minimum Inhibitory Concentration (MIC) and Minimum Bactericidal Concentration (MBC) for Twelve Antimicrobials (Biocides and Antibiotics) in Eight Strains of *Listeria monocytogenes*

**DOI:** 10.3390/biology11010046

**Published:** 2021-12-29

**Authors:** Cristina Rodríguez-Melcón, Carlos Alonso-Calleja, Camino García-Fernández, Javier Carballo, Rosa Capita

**Affiliations:** 1 Department of Food Hygiene and Technology, Veterinary Faculty, University of León, 24071 León, Spain; crodm@unileon.es (C.R.-M.); carlos.alonso.calleja@unileon.es (C.A.-C.); mc.garcia@unileon.es (C.G.-F.); 2 Institute of Food Science and Technology, University of León, 24071 León, Spain; 3 Food Technology Area, University of Vigo, 32004 Ourense, Spain; carbatec@uvigo.es

**Keywords:** *Listeria monocytogenes*, minimum inhibitory concentration, minimum bactericidal concentration, antibiotics, biocides

## Abstract

**Simple Summary:**

*Listeria monocytogenes* is the bacterium responsible for the majority of cases of human listeriosis, a foodborne infection that, in certain groups in the population (children, elderly, pregnant women and immunocompromised individuals), exhibits a high fatality rate (of up to 30%), and the need for hospital admission in more than 90% of cases. An awareness of the minimal concentrations for disinfectants and antibiotics necessary to destroy *L. monocytogenes*, may assist with choosing the most effective antimicrobials for controlling this microorganism, whether in the food industry or in the health system. The lethal concentrations of three disinfectants (sodium hypochlorite, benzalkonium chloride, and peracetic acid) and eight antibiotics (ampicillin, cephalothin, cefoxitin, erythromycin, chloramphenicol, gentamicin, tetracycline, vancomycin, and fosfomycin) for eight different strains of *L. monocytogenes* were determined in this research work. It was demonstrated that the lethal concentrations for the disinfectants tested were much lower than the concentrations customarily used of these compounds. The characteristics of the cell surface play an important role in the tolerance of *L. monocytogenes* to these biocides. A considerable prevalence of resistance to most of the antibiotics tested was noted, making it clear that the necessary measures to control resistance in *L. monocytogenes* must be adopted.

**Abstract:**

When selecting effective doses of antimicrobials, be they biocides or antibiotics, it is essential to know the minimum inhibitory concentrations (MICs) and minimum bactericidal concentrations (MBCs) of these substances. The present research determined the MICs and MBCs for three biocides, sodium hypochlorite (SH), benzalkonium chloride (BC), and peracetic acid (PAA), and nine antibiotics in eight strains of *Listeria monocytogenes* of varying serotypes. Marked intra-species differences were observed in the resistance of *L. monocytogenes* to the biocides and antibiotics. The MICs (ppm) for the biocides ranged between 1750 and 4500 for SH, 0.25 and 20.00 for BC, and 1050 and 1700 for PAA. Their MBCs (ppm) ranged from 2250 to 4500 for SH, 0.50 to 20.00 for BC, and 1150 to 1800 for PAA. The MICs (ppm) for antibiotics lay between 1 and 15 for ampicillin, 8 and 150 for cephalothin, 20 and 170 for cefoxitin, 0.05 and 0.20 for erythromycin, 4 and 50 for chloramphenicol, 3 and 100 for gentamicin, 2 and 15 for tetracycline, 2 and 80 for vancomycin, and 160 and 430 for fosfomycin. The corresponding MBCs (ppm) were from 5 to 20 for ampicillin, 9 to 160 for cephalothin, 70 to 200 for cefoxitin, 4 to 5 for erythromycin, 9 to 70 for chloramphenicol, 5 to 100 for gentamicin, 3 to 30 for tetracycline, 3 to 90 for vancomycin, and 160 to 450 for fosfomycin. Notably, erythromycin showed considerable efficacy, demonstrated by the low values for both MIC and MBC. Based on EUCAST and the CLSI criteria, all strains were susceptible to erythromycin. All strains were resistant to cephalothin, cefoxitin, gentamicin, and fosfomycin. Further values for resistance were 87.50% for ampicillin and vancomycin, 75.00% for tetracycline, and 62.50% for chloramphenicol. The high prevalence of antibiotic resistance is a matter for concern. A positive correlation was found between MIC and MBC values for most of the biocides and antibiotics. The higher the hydrophobicity of the cell surface, the higher the susceptibility to biocides, suggesting that surface characteristics of bacterial cells influence resistance to these compounds.

## 1. Introduction

Bacteria of the genus *Listeria* are short, Gram-positive non-spore-producing rods that have the ability to grow in a wide range of temperatures (0.5 °C to 45 °C), pH values (4.7 to 9.2), and osmotic pressures. These characteristics, along with the fact that they are facultative anaerobes, allow these microorganisms to survive under adverse environmental conditions [1].

A total of 26 species have so far been identified within the genus *Listeria* (Table 1). Of all these species the most prominent is *Listeria monocytogenes* because it causes the most cases of listeriosis, be it in humans or in animals. *Listeria ivanovii* also sometimes triggers listeriosis, and a few sporadic cases have been described where listeriosis was caused by *Listeria seeligeri* [2].

Listeriosis is a food-borne zoonosis that most frequently and most seriously affects the risk groups, collectively known as YOPIs (the Young, Old, Pregnant, and Immunocompromised). Invasive listeriosis is an infection associated with a high rate of hospital admissions and is the food-borne disease with the greatest lethality rate [20]. Moreover, this infection can give rise to grave harm or sequelae, such as meningitis, encephalitis, septicaemia, endocarditis, and miscarriages [21]. For these reasons, *L. monocytogenes* is a major risk for the food industry, and in particular for producers of ready-to-eat (RTE) foods [22]. Thus, several measures are applied to reduce the prevalence and/or the levels of this bacterium in RTE foodstuffs [23,24].

Among the disinfectants most widely used in food-processing environments are sodium hypochlorite (SH), benzalkonium chloride (BC), and peracetic acid (PAA). Chlorine-based disinfectants like SH are inexpensive oxidizing compounds that show powerful, broad-spectrum bactericidal activity [25]. For their part, compounds derived from quaternary ammonium, like BC, are cationic surfactants that act by destroying the lipid bilayer membrane and have an antimicrobial effect on several types of microorganisms [26]. The antimicrobial activity of PAA is also based on the oxidation of cell components [27]. Both SH and PAA are approved for various uses in the European Economic Area (EEA) and Switzerland, including as food and feed area disinfectants (Product-Type 4). This is subject to the specifications and conditions for use established by the Commission Implementing Regulation (EU) 2017/1273 in the case of SH, and the Commission Implementing Regulation (EU) 2016/672 for PAA. At present, the use of BC in a range of types of biocidal products, including disinfectants for areas where food and feed are processed, is being reviewed in the EEA and Switzerland [28].

If disinfectants are to be effective, they must be utilized in appropriate doses. Use at sub-lethal concentrations is not only ineffective but can even be counterproductive since low doses of biocides are linked to an increase in tolerance of these substances and resistance to antibiotics, in addition to a heightened bacterial capacity to form biofilm [28,29,30,31,32]. For this reason, awareness of the minimum inhibitory concentration (MIC) and minimum bactericidal concentration (MBC) of disinfectants is crucial.

In view of the seriousness of the infection, treatment with antibiotics is generally required for people suffering from invasive listeriosis. The increase in resistance to antibiotics over the last few decades has become a source of concern worldwide. Although various strategies are being devised to prevent and control this problem, bacterial resistance is becoming ever more frequent, both in clinical strains and in those found in the environment or in foodstuffs [33].

The aims of this study were: (1) to determine the minimum inhibitory concentrations (MICs) and minimum bactericidal concentrations (MBCs) for nine antibiotics of clinical interest and three biocides used in food-processing plants relative to eight strains of *L. monocytogenes* belonging to different serotypes; (2) to establish the relationship between the MIC and MBC values of the biocides and antibiotics; and (3) to know the influence of cell surface hydrophobicity on the susceptibility to antimicrobials.

## 2. Materials and Methods

### 2.1. Strains and Culturing Conditions

Eight strains of *L. monocytogenes* were used: ATCC (American Type Culture Collection) 19111 (serotype 1/2a), ATCC 19112 (serotype 1/2c), ATCC 19114 (serotype 4a), ATCC 19117 (serotype 4d), ATCC 13932 (serotype 4b), STCC (Spanish Type Culture Collection) 936 (serotype 1/2b), STCC 937 (serotype 3b), and STCC 938 (serotype 3c). The bacterial cultures were kept in storage at a temperature of −50 °C in tryptone soy broth (TSB; Oxoid Ltd., Hampshire, UK) with 20% (vol/vol) of glycerol. Prior to each experiment, aliquots of approximately 20 μL of the frozen culture were transferred to tubes containing 5 mL of TSB (Oxoid) that had been incubated overnight at 37 °C. Thereafter, the cultures were inoculated onto tryptone soy agar (TSA, Oxoid Ltd., Hampshire, UK) plates and stored at 4 °C until required for use.

### 2.2. Determination of the Minimum Inhibitory Concentration (MIC)

The MIC of the antimicrobials was determined by the method involving microdilution in culture broth, as indicated by the Clinical and Laboratory Standards Institute of the United States of America [34]. In this process, different concentrations of twelve antimicrobials, comprising three biocides and nine antibiotics, were used. The biocides tested were sodium hypochlorite (SH), benzalkonium chloride (BC), and peracetic acid (PAA). All three were obtained from the Sigma-Aldrich Co. (Saint Louis, MO, USA). In preparing the solutions required, the initial substance contained 10% free chlorine in the case of the SH, 95% (on the assumption the product was pure) for the BC, and 39% acetic acid equivalent for the PAA. Dehydrated antibiotics were purchased from the Sigma-Aldrich Co. (Saint Louis, MO, USA). They were ampicillin (AMP), cephalothin (KF), cefoxitin (FOX), erythromycin (E), chloramphenicol (C), gentamicin (CN), tetracycline (TE), vancomycin (VA), and fosfomycin (FOS). Before the start of each experiment, solutions of each of these compounds were prepared under aseptic conditions in sterile distilled water (FOX, CN, TE, VA, FOS), in 95% ethanol (E, C), in phosphate buffered saline (PBS) at pH 8.0 (AMP) or in PBS at pH 6.0 (KF). Three replicates were performed for each strain and antimicrobial compound.

Five colonies of each strain were taken from the TSA (Oxoid) plates, inoculated into 9 mL of TSB (Oxoid), and incubated at 37 °C for 18 to 24 h. In this experimental work, polystyrene microtiter plates with one hundred wells (Oy Growth Curves Ab Ltd., Helsinki, Finland) were used. The wells were filled with a total volume of 200 μL, made up of 20 μL of the antimicrobial solution at a range of concentrations and 180 μL of the third dilution of the inoculum to obtain a final concentration in the well of approximately 10^5^ cfu/mL. The concentration of the inoculum was confirmed by plating. Negative controls with 200 μL of TSB and 200 μL of the antimicrobial solutions and positive controls with 200 μL of the bacterial inoculum were used. Growth was determined by measuring the optical density of each sample in the range 480 to 520 nm (OD_480–520_) in a Bioscreen C MRB (Oy Growth Curves Ab). The value for MIC was set as the minimum concentration of the antimicrobial substance necessary to prevent bacterial growth after 48 h of incubation at 37 °C. The growth limit was deemed to be a value of 0.200 for OD_480–520_. Strains were classified as resistant, with reduced susceptibility (intermediate), or susceptible, based on given criteria. These were the guidelines set for *L. monocytogenes* in the case of AMP and E [35], the standards laid down for *Staphylococcus* spp. when considering C, TE, and FOS, for *Staphylococcus aureus* when considering CN and VA [35], and the norms used for *S. aureus* with respect to KF and FOX [36]. In certain cases, criteria established for another Gram-positive bacterium (*Staphylococcus* spp. or *S. aureus*) were employed because there were none for *L. monocytogenes*.

### 2.3. Determination of the Minimum Bactericidal Concentration (MBC)

The dilution in broth method was used to calculate the MBC for the antimicrobials [34]. A volume of 0.1 mL was removed from the wells in the microtiter plates (Oy Growth Curves Ab) where no growth was observed after 48 h of incubation at 37 °C, and was then inoculated onto the surface of TSA plates (Oxoid). They were incubated for 48 h at 37 °C, with MBC being taken to be the lowest concentration of the substance at which no colonies formed under these conditions. Since the limit of detection for this technique is 10 cfu/mL, the absence of any growth on a TSA plate indicated that the concentration lay below this value. The initial concentration of 10^5^ cfu/mL had thus been reduced to below 10 cfu/mL. Consequently, the MBC was effectively deemed to be the minimum concentration of antimicrobial capable of inactivating more than 99.99% of the bacteria present. Three replicates were performed for each strain and antimicrobial compound.

### 2.4. Determination of Cell Surface Hydrophobicity (CSH)

The CSH of strains was determined by the microbial adhesion to solvents (MATS) test based on affinity to non-polar solvents [37]. Hexadecane was used as the hydrocarbon phase. *L. monocytogenes* cells were grown in TSB for 24 h at 37 °C. Cells were harvested by centrifugation (4000 rpm, 10 min, 4 °C), washed twice with sterile PBS (Merck KGaA, Darmstadt, Germany), and re-suspended in TSB at an initial concentration of 10^5^ cfu/mL. After 24 h at 37 °C, the bacterial cells were centrifuged, washed twice with PBS, and re-suspended in 150 mM NaCl at a concentration of approximately 10^8^ cfu/mL. An aqueous-phase sample (0.4 mL) was obtained and absorbance at 405 nm was determined (Bioscreen C MBR, Oy Growth Curves Ab). The cell suspension (2.0 mL) was vortexed with 0.33 mL of hexadecane (Merck KGaA, Darmstadt, Germany) for 60 s and then allowed to stand for 15 min at room temperature, resulting in the complete separation of the two phases. The percentage of cells present in the solvent was calculated using the following equation: % affinity = 100 × [1 − (*A/A0*)], where *A0* is the absorbance of the original suspension at 405 nm prior to mixing, and *A* is the absorbance of the aqueous phase. Cell surface hydrophobicity was categorized as weak (<21%), moderate (21% to 50%), or strong (>50%) affinities [37]. All determinations were carried out eight times on four separate days (two replications were performed on the same day).

### 2.5. Statistical Analysis

A correlation analysis was performed to determine the relationship between the MIC and the MBC of the biocides and antibiotics and the percentages of affinity for hexadecane (hydrophobicity). The hydrophobicity values were compared for statistical significance using an analysis of variance techniques. Mean separations were obtained using Duncan’s multiple range test. Significant differences were established for a probability level of 5% (*p* < 0.05). All data processing in this study was carried out using the Statistica^®^ 8.0 software package (Statsoft Ltd., Tulsa, OK, USA).

## 3. Results

### 3.1. Minimum Inhibitory Concentration (MIC) and Minimum Bactricidal Concentration (MBC) for the Biocides

The MIC and MBC values for the three biocides tested on the eight strains of *L. monocytogenes* are shown in Table 2. SH was the substance requiring the greatest concentrations to inhibit the growth of strains after 48 h of incubation, with recorded MICs of between 1750 ppm (175 ppm of free chlorine) and 4500 ppm (450 ppm of free chlorine). The values noted for MBC were equal to or greater than those for MIC, ranging from 2250 ppm (225 ppm of free chlorine) to 4500 ppm (450 ppm of free chlorine).

The MICs for BC were the lowest among the three substances tested, ranging between 0.25 ppm and 20.00 ppm. As in the case of SH, the MBCs for BC were very similar to or only slightly higher than the MICs, ranging between 0.50 ppm and 20.00 ppm. With respect to PAA, the values recorded for MIC were slightly lower than those for SH, falling in the range of 1050 ppm to 1700 ppm. In all cases, the MBCs for PAA were slightly higher than the MICs for this substance, with values of between 1150 ppm and 1800 ppm.

### 3.2. Minimum Inhibitory Concentration (MIC) and Minimum Bactericidal Concentration (MBC) for Antibiotics

The values for the MICs and MBCs of nine antibiotics of clinical interest with regards to the eight strains of *L. monocytogenes* studied are presented in Table 3. The MICs varied notably depending on the combination of antibiotic and strain under consideration. The values (ppm) for MICs ranged from 1 to 15 for ampicillin, 8 to 150 for cephalothin, 20 to 170 for cefoxitin, 0.05 to 0.20 for erythromycin, 4 to 50 for chloramphenicol, 3 to 100 for gentamicin, 2 to 15 for tetracycline, 2 to 80 for vancomycin, and 160 to 430 for fosfomycin.

The values for MBCs were greater than or equal to those for MICs with respect to all of the strains tested. Moreover, considerable differences were observed between strains. The recorded values for MBCs (ppm) ranged between 5 and 20 for ampicillin, 9 and 160 for cephalothin, 70 and 200 for cefoxitin, 4 and 5 for erythromycin, 9 and 70 for chloramphenicol, 5 and 100 for gentamicin, 3 and 30 for tetracycline, 3 and 90 for vancomycin, and 160 and 450 for fosfomycin. Notably, erythromycin demonstrated considerable efficacy, as shown by the low values for MIC and MBC.

**Figure 1:**
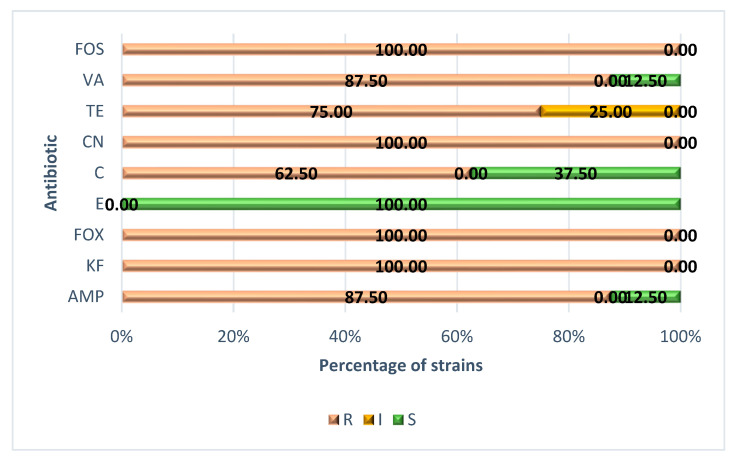
Percentage of strains of *Listeria monocytogenes* resistant (R), intermediate (I, with reduced susceptibility) or susceptible (S) to each of the nine antibiotics tested. AMP—ampicillin; KF—cephalothin; FOX—cefoxitin; E—erythromycin; C—chloramphenicol; CN—gentamicin; TE—tetracycline; VA—vancomycin; FOS—fosfomycin. LM—*Listeria monocytogenes*. American Type Culture Collection (ATCC) strains comprised ATCC 19111 (serotype 1/2a), ATCC 19112 (serotype 1/2c), ATCC 19114 (serotype 4a), ATCC 19117 (serotype 4d), and ATCC 13932 (serotype 4b). Those from the Spanish Type Culture Collection (STCC) comprised STCC 936 (serotype 1/2b), STCC 937 (serotype 3b), and STCC 938 (serotype 3c).

The percentages of strains that were resistant, intermediate, or susceptible to each of the antibiotics tested are shown in Figure 1. As can be seen, all of the strains presented resistance to cephalothin, cefoxitin, gentamicin, and fosfomycin. The prevalence of resistance was also high in the case of ampicillin and vancomycin (87.50%), tetracycline (75.00%), and chloramphenicol (62.50%). In contrast, all of the strains were susceptible to erythromycin.

**Figure 2:**
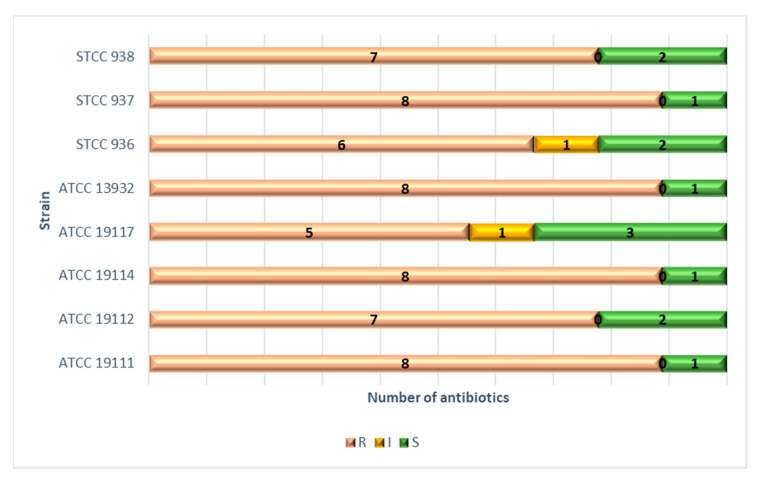
Number of antibiotics to which each strain of *L. monocytogenes* was resistant, intermediate (with reduced susceptibility), or susceptible. ATCC—American Type Culture Collection; STCC—Spanish Type Culture Collection.

Lastly, the number of antibiotics to which each of the strains was resistant is shown in Figure 2. Four strains (ATCC 19111, ATCC 19114, ATCC 13932, and STCC 937) were resistant to eight antibiotics, two strains (ATCC 19112 and STCC 938) to seven, one strain (STCC 936) to six, and one strain (ATCC 19117) to five different antibiotics.

### 3.3. Relationship between MICs and MBCs of Biocides and Antibiotics

Twenty-three positive correlations (34.8%; *p* < 0.05) and three negative correlations (4.5%; *p* < 0.05) were found among the 66 correlations tested (Table 4). In the case of MBCs (Table 5), 27 (40.9%) positive correlations and five (7.6%) negative correlations were found. A positive correlation (*p* < 0.001) was obtained between the MIC and the MBC values for each antimicrobial tested (Table 6).

### 3.4. Cell Surface Hydrophobicity

**Figure 3:**
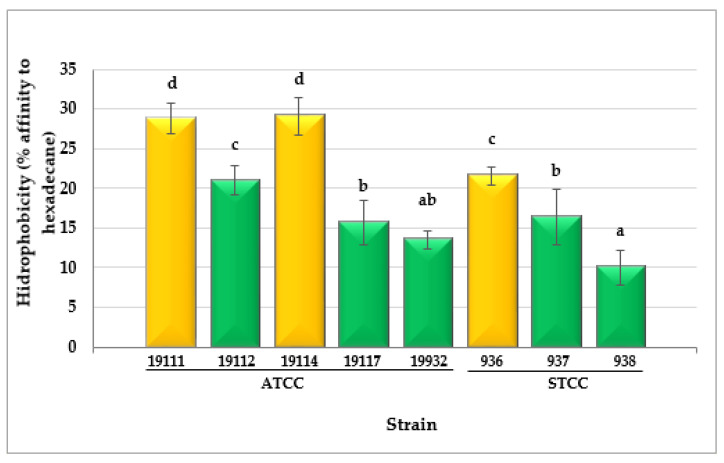
Cell surface hydrophobicity (CSH) values observed for cultures of eight *Listeria monocytogenes* strains. Data are means ± standard deviations (SD) for eight determinations. Mean values with no letters in common are significantly different (*p* < 0.05). ATCC—American Type Culture Collection; STCC—Spanish Type Culture Collection. The green columns represent weak CSH; the yellow columns represent moderate CSH.

Substantial differences (*p* < 0.001) were observed between the values for hydrophobicity in the various strains of *L. monocytogenes* tested. The percentage of affinity for hexadecane ranged from 10.04 ± 2.17% in the case of strain LM STCC 938 to 29.11 ± 2.36% for LM ATCC 19114 (Figure 3). The highest values for cell surface hydrophobicity were reflected by a higher percentage of cells moving to the hydrophobic phase of the MATS assay.

Three strains (37.5% of the total) had moderate cell surface hydrophobicity (between 21% and 50% affinity for hexadecane), whilst five strains (62.5%) showed weak reactions (<21% affinity for hexadecane). Notably, the hydrophobicity values correlated negatively with the MICs and MBCs of the biocides (significantly for SH and BC) (Table 7).

## 4. Discussion

### 4.1. Minimum Inhibitory Concentration (MIC) and Minimum Bactericidal Concentration (MBC) for Biocides

In previous works it has been demonstrated that contact with sub-inhibitory doses of various biocides habitually used in the food industry can trigger the adaptation of bacteria to the substances in question, as well as encourage the emergence of resistance to antibiotics and an increased ability to form biofilm [28,30,31,32,38,39]. This constitutes a challenge for food safety and public health. Thus, when establishing effective disinfection protocols, it is necessary to be aware of the MICs and MBCs of disinfectants in relation to the various microorganisms that may be present in food-processing environments. The current study determined the MICs and MBCs for three biocides widely used in the food industry, sodium hypochlorite (SH), benzalkonium chloride (BC), and peracetic acid (PAA), when applied to eight strains of *L. monocytogenes* of differing serotypes.

The disinfectant requiring the greatest concentrations as MICs was SH, needing between 1750 and 4500 ppm, which equates to 175 to 450 ppm of free chlorine. These values fall within the range observed by Lundén et al. [40], who quoted a value of 2500 ppm, and by Rodríguez-Melcón et al. [32], who recorded the value of 3500 ppm, both relating to strains of *L. monocytogenes*. A similar MIC of 5000 ppm was also noted by Buzón-Durán et al. [29] with respect to strains of another Gram-positive microorganism, specifically methicillin-resistant *Staphylococcus aureus* (MRSA). It should be noted that the comparison of our results with those of other research works should be carried out with caution, since the composition of the culture broth used for the determination of MIC and MBC (TSB in the study reported here) could influence the results obtained [41,42].

Benzalkonium chloride, a compound belonging to the group of derivatives of quaternary ammonium, was the disinfectant that produced inhibition at the lowest concentrations (between 0.25 ppm and 20.00 ppm). These values are similar to those noted by other authors for Gram-positive bacteria, such as the 2 ppm recorded by Buzón-Durán et al. [29] for MRSA and the range of between 3 and 13 ppm for different strains of *L. monocytogenes* reported by Rodríguez-Melcón et al. [32]. In the case of Gram-negative bacteria, results in line with those of the present research work have also been recorded, with values of 8 ppm for *Salmonella enterica* serotype Typhimurium [39], 15 ppm for *Cronobacter sakazakii,* and 20 ppm for *Yersinia enterocolitica* [28].

The MIC values for PAA observed in this study, ranging from 1050 ppm to 1700 ppm of 39% PAA, which equates approximately to 410 ppm and 660 ppm of the pure product, were higher than the MICs recorded for peroxyacids in previous research on *L. monocytogenes*, which fell in the range of 100 to 110 ppm [43]. The differences in the results of the various reports may be due to the fact that not all strains present the same susceptibility to biocides, as has been demonstrated previously [32]. Moreover, the varying composition of the mixtures of peroxyacids may also have been responsible for the marked differences in the results obtained by the various authors cited [44]. It must be pointed out that the MICs noted in the current work are in line with the values recorded for PAA in previous work on certain Gram-negative bacteria, where a value of 1200 ppm, equivalent to 468 ppm of the pure substance, was observed as the MIC for *Cronobacter sakazakii*, and a value of 1275 ppm, equating to 497.3 ppm of the product in its pure state, was observed as the MIC for *Yersinia enterocolitica* [28].

Unlike with antibiotics, no concentrations of disinfectants could be specified that allowed the bacteria to be classified as resistant, intermediate, or susceptible to these compounds. Various authors considered bacteria to be resistant when their MIC values are at least two to four times higher than those found in more susceptible strains [45]. Following this criterion, work done by Rodríguez-Melcón et al. [32] established two populations of strains of *L. monocytogenes* in terms of their susceptibility to BC. These were susceptible strains, with an average value of 3 ppm as the MIC, and resistant strains, where the values for the MIC were equal to or greater than 9 ppm. In the present research, strains of *L. monocytogenes* may be classified relative to SH as forming two groups, sensitive, with the MIC falling in the range of 1750 to 2250 ppm, and resistant, where the corresponding values were between 3500 and 4500 ppm. This could be similarly applied to BC, with sensitive strains of MICs ranging from 0.25 to 4 ppm, and resistant strains ranging from 19 to 20 ppm. Regarding PAA, the differences among strains were less marked and did not allow for a classification of this type.

In relation to MBCs, the values obtained for SH, ranging from 2250 ppm to 4500 ppm, coincide with the findings of earlier investigations [32], where the values observed were between 3500 ppm and 4500 ppm. Along these lines, it must also be kept in mind that the values for MBC may vary as a function of the growth mode of the bacteria. Thus, several studies, including Smith and Hunter [46], have highlighted the fact that MBCs for microorganisms like MRSA or *Pseudomonas aeruginosa* are between 10 and 1000 times higher for sessile bacteria forming part of biofilms than for planktonic bacteria (free-living).

It should be pointed out that the disinfectants tested are habitually used in concentrations much higher than their MBCs to achieve rapid, effective inactivation of microorganisms, making it highly unlikely for bacteria to survive and develop resistance under normal conditions [47]. The concentrations of free chlorine usually employed in the case of substances releasing chlorine such as SH are in the order of 800 to 2000 ppm of free chlorine [26,48]. They range between 1000 and 5000 ppm for quaternary ammonium compounds like benzalkonium chloride [26,49,50]. In the case of PAA, the values are 10,000 ppm to 150,000 ppm of the pure substance [51,52]. However, it is a known fact that under certain circumstances sub-lethal exposure to biocides does occur. This can be the outcome of incorrect calculations of the dosage to be used, inappropriate storage of disinfectants, an uneven distribution of the substances in use, or the presence of excessive amounts of residues of organic materials, which neutralize different biocidal substances, such as sodium hypochlorite [30]. Situations of this kind should be avoided because, as commented above, exposure of the bacteria present in food industry plants and equipment to sub-lethal doses of biocides poses a challenge to food safety since it favours adaptation to disinfectants, thus increasing the risk of the emergence of resistance to antibiotics and the capacity to form biofilm [28].

### 4.2. Minimum Inhibitory Concentration (MIC) and Minimum Bactericidal Concentration (MBC) for Antibiotics

Over recent decades there has been a marked increase in the prevalence of bacteria resistant to antibiotics, which is emerging as one of the principal threats to public health worldwide [33]. It is estimated that by 2050 infections by resistant bacteria will have become the prime cause of mortality around the world, leading to some ten million deaths annually, surpassing the values for cardiovascular disease and cancer. This estimated number of deaths foreseen within three decades contrasts with the 700,000 fatalities attributable to bacteria in 2014 [53]. Moreover, resistance to antibiotics has major financial repercussions since it is estimated that infections by these bacteria cost the health systems of the European Union and European Economic Area (EEA) approximately 1.1 thousand million euros every year [54].

In the work reported here, the prevalence of resistance depended on the antibiotic in question. Considerable percentages of strains, between 62.5% and 100%, were resistant to all the antibiotics tested, the sole exception being erythromycin, to which all the strains were susceptible. It is especially worrying that high levels of resistance were seen for ampicillin, chloramphenicol, gentamicin, tetracycline, and vancomycin, which are antibiotics used to treat invasive listeriosis. Beta-lactams are the antibiotics of choice for such treatments, principally ampicillin, administered alone or in combination with gentamicin. In the case of allergy to beta-lactams, it is customary to administer erythromycin, vancomycin, trimethoprim/sulfamethoxazole, or fluoroquinolones. Vancomycin is also used for treating listeriosis during pregnancy [55]. Other antibiotics that are sometimes used to treat this infection include rifampicin, tetracycline, and chloramphenicol [55]. It should be noted that resistance to the antibiotics indicated has been previously highlighted in strains of *L. monocytogenes* of different origins [56,57,58,59,60].

Furthermore, it must be stressed that the antibiotics to which the strains were resistant are hugely important in both human and veterinary medicine. The antibiotics ampicillin, cephalothin, cefoxitin, erythromycin, gentamicin, vancomycin, and fosfomycin are deemed “critically important”, while chloramphenicol and tetracycline are considered “highly important” in human medicine, according to the World Health Organization [61]. The World Organization for Animal Health [62] classifies ampicillin, erythromycin, gentamicin, and tetracycline as “critically important”, and cephalothin and fosfomycin as “highly important” in terms of veterinary medicine. In view of the clinical importance of these antibiotics, the considerable prevalence of resistance found is a cause for concern, including when antibiotics not directly used to treat listeriosis are affected, because of the possibility that resistance genes may be transferred horizontally to other genera of pathogenic bacteria [33].

Strains of *L. monocytogenes* were previously susceptible to most of the antibiotics effective against Gram-positive bacteria. In a research work carried out more than three decades ago by Wiggins et al. [63], the MIC values for ampicillin, penicillin, erythromycin, and tetracycline for 175 strains of *L. monocytogenes* were reported to be below the cut-off point established by the CLSI. However, in recent years a considerable increase has been observed in the prevalence of resistance in bacteria of this microbial species [57,64].

The selective pressure exerted by the use of antibiotics, particularly when incorrectly employed at sub-inhibitory doses, has been identified as the principal cause of the marked growth in the prevalence of resistance in recent decades [33,65]. Moreover, several recent works have highlighted the fact that changes in profiles of resistance to antibiotics may be due to the exposure of microorganisms to sub-inhibitory concentrations of biocides or other sub-lethal stressing factors [28,30,33,66]. Furthermore, the possibility of the horizontal transfer of mobile genetic elements, such as transposons or plasmids, between bacteria of the same or different genera, facilitates a rapid spread of resistance genes. This too is among the causes of the increase in the prevalence of resistance to antibiotics observed in recent decades [67].

### 4.3. Relationship between MICs and MBCs of Biocides and Antibiotics

Positive correlations were observed for the MICs and the MBCs between different classes of antimicrobials. Thus, a total of seven classes of antibiotics were used, including beta-lactams (ampicillin, cephalothin, and cefoxitin), macrolides (erythromycin), phenicols (chloramphenicol), aminoglycosides (gentamicin), tetracyclines (tetracycline), glycopeptides (vancomycin), and fosfomycin. The fact that such compounds have unrelated modes of action and mechanisms of resistance [68] suggest that different genes involved in antibiotic resistance are carried in the same mobile genetic elements, as previously reported [69]. Thus, it has been demonstrated that co-selection and co-transfer are a common phenomenon in antibiotic resistance emergence and spread [33].

### 4.4. Cell Surface Hydrophobicity

The values for hydrophobicity obtained for the various strains of *L. monocytogenes* (10.04 ± 2.17% to 29.11 ± 2.36%) fall in the range of previous studies (4.84 ± 1.11% to 31.82 ± 5.98%) using xylene as the hydrocarbon phase [70]. The negative correlations found between the hydrophobicity and the MICs or MBCs of the biocides should be noted. The higher the hydrophobicity, the higher the susceptibility to the biocides. These results reveal that cell surface plays an important role in the tolerance of *L. monocytogenes* to these antimicrobials, and especially to SH and BC. The relationship between high hydrophobicity and high susceptibility to hydrophobic antimicrobials (e. g. benzalkonium chloride or erythromycin) has been observed by other authors [71,72]. By contrast, these results do not coincide with several research works performed with Gram-negative bacteria, where bacterial cells with a high cell surface hydrophobicity have shown an increased tolerance to biocides [73], which is a consequence of the low number of charged (hydrophilic) binding sites for the biocides [30,74].

## 5. Conclusions

It was demonstrated that the MICs and MBCs for the biocides tested, sodium hypochlorite, benzalkonium chloride, and peracetic acid, relative to *L. monocytogenes*, were much lower than the concentrations of these disinfectants customarily used. For these compounds to be completely efficacious, they must exceed MBCs in all of the areas treated, with checks on aspects such as the correct calculation of the concentrations to be employed, even distribution of the disinfecting substances, and prior elimination of any residues of organic matter, the latter being of particular importance when chlorinated compounds are in use. A positive relationship was found between cell surface hydrophobicity and susceptibility to biocides, indicating that the characteristics of the cell surface play an important role in the tolerance of *L. monocytogenes* to these compounds.

A considerable prevalence of resistance to most of the antibiotics tested was noted, making it clear that the necessary measures to control resistance in *L. monocytogenes* must be adopted. Of the nine antibiotics included in the study, erythromycin had the greatest antimicrobial efficacy, since it had the lowest values for both MIC and MBC. The positive correlations observed for the MICs and MBCs between the biocides and the antibiotics with different modes of action suggest that resistance genes are carried in the same mobile genetic elements.

An awareness of the MICs and MBCs for biocides and antibiotics against *L. monocytogenes* may assist with choosing the most effective antimicrobials for controlling this microorganism, whether in the food industry or in the health system. Nevertheless, the marked intra-species differences observed make it clear that including various strains in any studies aimed at determining the resistance of *L. monocytogenes* to biocides and antibiotics is vital.

## Figures and Tables

**Table 1:** Species in the genus *Listeria*. Adapted from Nwaiwu [3].

Species	Year of Description	Reference
*Listeria monocytogenes*	1940	[4]
*Listeria innocua*	1983	[5]
*Listeria seeligeri*	1983	[6]
*Listeria welshimeri*	1983	[6]
*Listeria ivanovii*	1984	[7]
*Listeria grayi*	1992	[8]
*Listeria marthii*	2010	[9]
*Listeria rocourtiae*	2010	[10]
*Listeria fleischmannii*	2013	[11]
*Listeria weihenstephanensis*	2013	[12]
*Listeria aquatica*	2014	[13]
*Listeria cornellensis*	2014	[13]
*Listeria floridensis*	2014	[13]
*Listeria grandensis*	2014	[13]
*Listeria riparia*	2014	[13]
*Listeria booriae*	2015	[14]
*Listeria newyorkensis*	2015	[14]
*Listeria goaensis*	2018	[15]
*Listeria costaricensis*	2018	[16]
*Listeria thailandensis*	2019	[17]
*Listeria valentina*	2020	[18]
*Listeria cossartiae*	2021	[19]
*Listeria farberi*	2021	[19]
*Listeria immobilis*	2021	[19]
*Listeria portnoyi*	2021	[19]
*Listeria rustica*	2021	[19]

**Table 2:** Minimum inhibitory concentration (MIC; ppm) and minimum bactericidal concentration (MBC; ppm) for three biocides on eight strains of *Listeria monocytogenes*.

Strain	Biocide		
	SH	BC	PAA
ATCC 19111	1750 (2250)	0.25 (1.50)	1650 (1800)
ATCC 19112	2250 (2250)	0.50 (0.50)	1500 (1550)
ATCC 19114	3500 (3900)	2.00 (4.00)	1050 (1150)
ATCC 19117	3500 (3500)	0.75 (3.00)	1700 (1750)
ATCC 13932	3500 (3700)	4.00 (5.00)	1100 (1250)
STCC 936	3500 (3900)	3.00 (5.00)	1050 (1150)
STCC 937	4000 (4500)	20.00 (20.00)	1600 (1650)
STCC 938	4500 (4500)	19.00 (19.00)	1400 (1600)

SH—sodium hypochlorite; BC—benzalkonium chloride; PAA—peracetic acid. LM—*Listeria monocytogenes*. ATCC—American Type Culture Collection; STCC—Spanish Type Culture Collection. The values not in brackets correspond to the minimum inhibitory concentration (MIC), whilst bracketed values indicate the minimum bactericidal concentration (MBC).

**Table 3:** Minimum inhibitory concentration (MIC; ppm) and minimum bactericidal concentration (MBC; ppm) for nine antibiotics of clinical interest on eight strains of *Listeria monocytogenes*.

Strain	Antibiotic								
	AMP	KF	FOX	E	C	CN	TE	VA	FOS
ATCC 19111	7 (10)	150 (150)	170 (180)	0.05 (4)	20 (70)	45 (50)	15 (30)	80 (90)	350 (350)
ATCC 19112	11 (11)	140 (140)	160 (170)	0.10 (5)	4 (10)	45 (45)	14 (18)	45 (50)	160 (160)
ATCC 19114	9 (9)	80 (90)	140 (180)	0.05 (5)	20 (70)	20 (20)	8 (11)	20 (20)	230 (290)
ATCC 19117	1 (5)	8 (20)	150 (200)	0.20 (5)	20 (55)	4 (15)	2 (10)	2 (3)	430 (450)
ATCC 13932	9 (9)	70 (70)	160 (160)	0.20 (5)	35 (60)	20 (20)	11 (11)	20 (20)	250 (260)
STCC 936	5 (6)	8 (9)	20 (70)	0.20 (5)	5 (30)	3 (5)	2 (3)	20 (20)	170 (180)
STCC 937	15 (15)	150 (150)	170 (200)	0.20 (5)	50 (70)	50 (50)	15 (30)	35 (40)	240 (280)
STCC 938	13 (20)	150 (160)	150 (150)	0.20 (5)	5 (9)	100 (100)	15 (30)	50 (60)	220 (230)
CUT-OFF POINTS S ≤ - R >	1–1	0.12–0.50 *	4–8	1–1	8–8	1–1	1–2	2–2	32–32

AMP—ampicillin; KF—cephalothin; FOX—cefoxitin; E—erythromycin; C—chloramphenicol; CN—gentamicin; TE—tetracycline; VA—vancomycin; FOS—fosfomycin. LM—*Listeria monocytogenes*. ATCC—American Type Culture Collection; STCC—Spanish Type Culture Collection. Values not in brackets correspond to minimum inhibitory concentrations (MICs), and those in brackets to minimum bactericidal concentrations (MBC). The cut-off points for MIC used to classify strains as susceptible (MIC ≤ lower cut-off), of reduced susceptibility (MIC > lower cut-off and ≤ upper cut-off), and resistant (MIC > upper cut-off) are indicated. The criteria for AMP and E were specifically for *L. monocytogenes* [35]. Those used for C, TE, and FOS were initially intended for *Staphylococcus* spp. [35], those used for CN and VA were intended for *S. aureus* [35] and those used for KF and FOX were also intended for *S. aureus* [36]. *—quality control range. Green shading shows susceptible strains, yellow shading shows strains with reduced susceptibility, and red shading indicates strains that are resistant, in accordance with the criteria applied.

**Table 4:** Coefficients of correlation between the MIC values of 12 biocides and antibiotics in eight strains of *Listeria monocytogenes*.

	Biocides (MIC)			Antibiotics (MIC)								
	SH	BC	PAA	AMP	KF	FOX	E	C	CN	TE	VA	FOS
SH	-											
BC	0.715 ***	-										
PAA	−0.284	0.144	-									
AMP	0.273	0.734 ***	−0.016	-								
KF	−0.182	0.483 *	0.385	0.809 ***	-							
FOX	−0.169	0.185	0.568 **	0.387	0.640 ***	-						
E	0.706 ***	0.507 *	−0.018	0.000	−0.348	−0.265	-					
C	0.196	0.348	0.164	0.292	0.147	0.451 *	0.194	-				
CN	0.220	0.696 ***	0.297	0.732 ***	0.829 ***	0.438 *	0.006	−0.119	-			
TE	−0.166	0.471 *	0.319	0.818 ***	0.977 ***	0.678 ***	−0.268	0.229	0.801 ***	-		
VA	−0.513 *	0.135	0.367	0.412 *	0.808 ***	0.342	−0.492 *	0.141	0.646 ***	0.774 ***	-	
FOS	−0.155	−0.235	0.590 **	−0.576 **	−0.230	0.380	−0.011	0.271	−0.236	−0.231	−0.080	-

SH—sodium hypochlorite; BC—benzalkonium chloride; PAA—peracetic acid; AMP—ampicillin; KF—cephalothin; FOX—cefoxitin; E—erythromycin; C—chloramphenicol; CN—gentamicin; TE—tetracycline; VA—vancomycin; FOS—fosfomycin; HYD—hydrophobicity, microbial adhesion to solvents (MATS) assay was used using hexadecane as hydrocarbon phase. ***— *p* < 0.001; **— *p* < 0.01; *— *p* < 0.05.

**Table 5:** Coefficients of correlation between the MBC values of 12 biocides and antibiotics in eight strains of *Listeria monocytogenes*.

	Biocides (MBC)			Antibiotics (MBC)								
	SH	BC	PAA	AMP	KF	FOX	E	C	CN	TE	VA	FOS
SH	-											
BC	0.785 ***	-										
PAA	−0.312	0.171	-									
AMP	0.374	0.798 ***	0.289	-								
KF	−0.128	0.435*	0.471 *	0.821 ***	-							
FOX	0.144	0.079	0.598 **	0.162	0.430 *	-						
E	0.601 **	0.301	−0.475 *	0.052	−0.341	−0.157	-					
C	0.047	−0.094	0.027	−0.342	−0.104	0.479 *	−0.354	-				
CN	0.140	0.631 ***	0.506 *	0.937 ***	0.833 ***	0.209	−0.158	−0.414 *	-			
TE	−0.017	0.566 **	0.698 ***	0.799 ***	0.913 ***	0.473 *	−0.453 *	0.017	0.854 ***	-		
VA	−0.426 *	0.126	0.517 **	0.549 **	0.807 ***	0.108	−0.749 ***	−0.127	0.694 ***	0.805 ***	-	
FOS	−0.043	−0.132	0.510 *	−0.325	−0.210	0.618 **	−0.325	0.633 ***	−0.178	0.063	−0.156	-

SH—sodium hypochlorite; BC—benzalkonium chloride; PAA—peracetic acid; AMP—ampicillin; KF—cephalothin; FOX—cefoxitin; E—erythromycin; C—chloramphenicol; CN—gentamicin; TE—tetracycline; VA—vancomycin; FOS—fosfomycin. ***— *p* < 0.001; **— *p* < 0.01; *— *p* < 0.05.

**Table 6:** Coefficients of correlation between MIC and MBC values of 12 antimicrobials in eight strains of *Listeria monocytogenes*.

Biocides			Antibiotics								
SH	BC	PAA	AMP	KF	FOX	E	C	CN	TE	VA	FOS
0.967 ***	0.995 ***	0.979 ***	0.850 ***	0.996 ***	0.905 ***	0.571 **	0.767 ***	0.993 ***	0.868 ***	0.997 ***	0.974 ***

Values are the coefficient of correlation between the MIC and the MBC for each antimicrobial. SH—sodium hypochlorite; BC—benzalkonium chloride—PAA, peracetic acid; AMP—ampicillin; KF—cephalothin; FOX—cefoxitin; E—erythromycin; C—chloramphenicol; CN—gentamicin; TE—tetracycline; VA—vancomycin; FOS—fosfomycin. ***—*p* < 0.001; **—*p* < 0.01.

**Table 7:** Coefficients of correlation between hydrophobicity and MIC or MBC values of twelve biocides and antibiotics in eight strains of *Listeria monocytogenes*.

	Biocides			Antibiotics								
	SH	BC	PAA	AMP	KF	FOX	E	C	CN	TE	VA	FOS
HYD	−0.504 *	−0.580 **	−0.389	0.017	0.116	0.103	−0.725 ***	−0.365	−0.198	0.117	0.140	−0.328
	(−0.532 **)	(−0.616 **)	(−0.387)	(−0.186)	(0.113)	(0.006)	(−0.082)	(−0.097)	(−0.155)	(−0.237)	(0.099)	(−0.313)

SH—sodium hypochlorite; BC—benzalkonium chloride; PAA—peracetic acid; AMP—ampicillin; KF—cephalothin; FOX—cefoxitin; E—erythromycin; C—chloramphenicol; CN—gentamicin; TE—tetracycline; VA—vancomycin; FOS—fosfomycin; HYD—hydrophobicity, microbial adhesion to solvents (MATS) assay was used using hexadecane as hydrocarbon phase. ***—*p* < 0.001; **—p < 0.01; *—*p* < 0.05. Values without brackets (first row) represent the correlations between hydrophobicity and MIC values. Values in brackets (second row) represent the correlations between hydrophobicity and MBC values.

## Data Availability

Not applicable.
